# The association of enteric neuropathy with gut phenotypes in acute and progressive models of Parkinson’s disease

**DOI:** 10.1038/s41598-021-86917-5

**Published:** 2021-04-12

**Authors:** Rachel M. McQuade, Lewis M. Singleton, Hongyi Wu, Sophie Lee, Remy Constable, Madeleine Di Natale, Mitchell T. Ringuet, Joel P. Berger, Jessica Kauhausen, Clare L. Parish, David I. Finkelstein, John B. Furness, Shanti Diwakarla

**Affiliations:** 1 Department of Medicine, Western Health, Melbourne University, Sunshine, VIC 3021 Australia; 2 College of Health and Biomedicine, Victoria University, Sunshine, VIC 3021 Australia; 3 Digestive Physiology and Nutrition Laboratory, The Florey Institute of Neuroscience and Mental Health, Parkville, VIC 3010 Australia; 4 Department of Anatomy and Neuroscience, University of Melbourne, Parkville, VIC 3010 Australia; 5 JP Berger Consulting, Boston, MA 02111 USA; 6 Stem Cells and Neural Development Laboratory, The Florey Institute of Neuroscience and Mental Health, Parkville, VIC 3010 Australia; 7 Parkinson’s Disease Laboratory, The Florey Institute of Neuroscience and Mental Health, Parkville, VIC 3010 Australia

**Keywords:** Enteric nervous system, Gastrointestinal system

## Abstract

Parkinson’s disease (PD) is associated with neuronal damage in the brain and gut. This work compares changes in the enteric nervous system (ENS) of commonly used mouse models of PD that exhibit central neuropathy and a gut phenotype. Enteric neuropathy was assessed in five mouse models: peripheral injection of MPTP; intracerebral injection of 6-OHDA; oral rotenone; and mice transgenic for A53T variant human α-synuclein with and without rotenone. Changes in the ENS of the colon were quantified using pan-neuronal marker, Hu, and neuronal nitric oxide synthase (nNOS) and were correlated with GI function. MPTP had no effect on the number of Hu+ neurons but was associated with an increase in Hu+ nuclear translocation (P < 0.04). 6-OHDA lesioned mice had significantly fewer Hu+ neurons/ganglion (P < 0.02) and a reduced proportion of nNOS+ neurons in colon (P < 0.001). A53T mice had significantly fewer Hu+ neurons/area (P < 0.001) and exhibited larger soma size (P < 0.03). Treatment with rotenone reduced the number of Hu+ cells/mm^2^ in WT mice (P < 0.006) and increased the proportion of Hu+ translocated cells in both WT (P < 0.02) and A53T mice (P < 0.04). All PD models exhibited a degree of enteric neuropathy, the extent and type of damage to the ENS, however, was dependent on the model.

## Introduction

PD is a progressive neurodegenerative disorder that involves lesion of subcortical nuclei of the basal ganglia. Characterized by intracellular aggregation of alpha synuclein (α-syn) and loss of dopaminergic neurons, PD affects the substantia nigra pars compacta (SNpc), critical for motor function^1^, and other nuclei^2^. Consequently, PD patients experience motor disturbances including rigidity, akinesia, bradykinesia, postural instability and resting tremor^3^. In addition to these motor symptoms, most PD patients develop some form of autonomic disorder involving the cardiovascular, genitourinary, thermoregulatory and gastrointestinal (GI) systems^4^. GI dysfunction is highly prevalent, affecting 80–90% of PD patients, and includes symptoms such as dysphagia (difficulty swallowing), gastroparesis (slowed gastric emptying) and chronic constipation. Interestingly, these GI symptoms often precede the onset of motor deficits by decades^5^.

GI function is primarily controlled by the enteric nervous system (ENS), a subdivision of the autonomic nervous system, and its central nervous system (CNS) connections^6^. Beginning in the oesophagus and extending down to the anus, the ENS is embedded in the lining of the GI tract and interacts with the CNS through the vagus and pelvic nerves as well as sympathetic (prevertebral ganglia) connections that regulate contraction, relaxation, secretion and absorption throughout the GI tract^7^. Studies focusing on the ENS have found aggregated α­syn in the GI tract of almost every patient with PD; in some cases, the presence of α­syn in the ENS has been detected up to 20 years prior to diagnosis^5,8^. Neuronal loss and neurochemical changes have also been observed in the ENS of patients with PD, and a positive correlation between gut permeability and intestinal levels of aggregated α-syn has been observed in humans^9,10^.

Whilst in PD patients GI dysfunction and ENS pathology is now well recognised, the onset, severity, and role of the ENS in GI dysfunction in animal models of PD is contentious. Conflicting results exist regarding whole gut transit time and stool frequency in the α-syn transgenic mouse model (which expresses the human A53T mutation), with delayed whole gut transit time and reduced fecal pellet output reported as early at 3 months of age in some studies^11,12^, whilst other studies have failed to detect differences as late as 15 months of age^13,14^.

The onset of enteric neuropathy and GI dysfunction is also variable in chemical models of PD including 1-methyl-4-phenyl-1,2,3,6-tetrahydropyridine (MPTP), 6-hydroxydopamine (6-OHDA) and rotenone. While some studies have demonstrated a reduced stool frequency following MPTP^15,16^, others have shown a rapid transient increase but no long term change^17^. Conflicting findings have also been observed for TH and dopamine expression in the gut of 6-OHDA rodent models, describing both increased and decreased levels^18,19^.

The extent to which degeneration in the ENS is correlated with GI dysfunction in various models of PD remains a source of confusion. This lack of consistency in reported GI symptoms across animal models of PD has hampered our understanding of the role of the ENS in the pathophysiology of PD and is likely a contributing factor in the slow development of treatments for GI dysfunction in PD patients^17,20^.

In this study, we investigated and directly compared mouse models of PD for enteric neuropathy (assessing neuronal number/ganglia, cell size, proportion of nNOS neurons, Hu nuclear translocated in the myenteric plexus) and correlated these findings with GI dysfunction (assessed via bead expulsion, fecal pellet output (FPO) and fecal water content (FWC)). We hypothesize that the level of enteric neuropathy will vary across animal models and that GI symptoms and enteric neuropathy will be most pronounced in A53T mice treated with rotenone.

## Methods

### Animals

All procedures involving mice conformed to the Australian National Health and Medical Research Council (NHMRC) code of practice for the care and use of animals for scientific purposes and were approved by the Florey Institute of Neuroscience and Mental Health Animal Ethics Committee. Swiss, C57bl/6 and A53T mice were group housed (2–5 animals/cage) in a temperature- and humidity-controlled room under a 12-h light/dark cycle. Food and water were available ad libitum. Mice (B6;C3-Tg-Prnp/SNCA*A53T/83Vle/J) were originally obtained in breeding pairs from Jackson Laboratories (Bar Harbor, ME). A colony of transgenic mice that carry the human A53T mutation driven by the mouse prion promoter was established from A53T heterozygous breeders to produce both wild-type (WT) and homozygous Tg (A53T) mice, as previously described^14^.

### 6-OHDA lesioning

Using previously established methods^21^, female swiss mice, aged 16 weeks (n = 5 sham, n = 7 6OHDA lesioned) were anesthetized via 2–5% isoflurane inhalation (Baxter; Deerfield, IL, USA), their heads secured in a stereotaxic head frame and analgesia administered perioperatively (meloxicam, 2 mg/kg, i.p). A 1.5 μg/μl solution of 6-OHDA (Sigma) was prepared in saline containing ascorbic acid (0.2 mg/ml) and kept on ice until the time of injection. A 10 μl glass syringe (with a 26-gauge needle) mounted in a syringe pump (Cole-Parmer, Vernon Hills, IL) was inserted into the right SNpc through a small hole drilled through the top of the skull. A single injection (1.5 μl, delivered at a rate of 1 μl/min) of 6-OHDA (3 µg) was made into the right SNpc (3.0 mm posterior and 1.05 mm lateral to Bregma, and 4.7 mm ventral to the dura surface). On completion of the injection, the needle was left in place for 2 min before withdrawal. After surgery, the skin was sutured, antiseptic (1% w/w iodine, Betadine; Faulding and Company, Salisbury, South Australia) was applied to the wound, and the mice were left in a warmed cage to recover.

Using the same surgical procedures, sham lesioned mice received an intranigral injection of saline containing ascorbic acid (0.2 mg/ml). Three days after surgery, mice underwent amphetamine-induced rotational testing to confirm sufficient ablation of the midbrain dopamine neurons, as previously described^22^. In brief, mice were injected with D- amphetamine (0.25 mg/kg, i.p, Tocris Bioscience) and placed in an automated rotometer. After 10 min, net rotations over a 60 min period were recorded. At 2 weeks after lesioning fecal pellet output and colonic bead expulsion were assessed in each mouse before euthanasia. This dose and time-frame was chosen based on previously published literature showing significant TH and Neun positive cell loss in mouse SNpc, and motor deficits at 2 weeks post-lesion^23,24^. A summary of the treatment regimens utilized in this study is included in Table 1.

**Table 1:** Animal model details.

Model	Mice	Gender	Age	Dose & Duration	Route	Details
6-OHDA	Swiss mice	Female	Aged 16 weeks	Single injection (3 µg)	Unilateral SNpc injection	Killed at 2 weeks post-treatment, 18 weeks of age (n = 5 sham, n = 7 6OHDA lesioned)
MPTP	C57bl/6 mice	Male	Aged 16 weeks	4 equal doses, each two hours apart (total dose 45 mg/kg)	Intraperitoneal injection	Killed at 3 weeks post-treatment, 19 weeks of age (n = 15 saline-treated mice, n = 16 MPTP-treated mice)
Rotenone	WT and A53T mice	Female	Aged 12 weeks	Daily dose 30 (mg/kg) every day, for 28 days	Oral gavage	Killed at 16 weeks post-treatment, 32 weeks of age (n = 21 WT and A53T n = 8)

### MPTP lesioning

MPTP lesioning of mice was performed as previously described^16,25^. Briefly, 16-week old male C57B/6 mice (n = 15 saline-treated mice, n = 16 MPTP-treated mice) were injected intraperitoneally with 45 mg/kg MPTP (Sigma-Aldrich) in saline, delivered in 4 doses, each two hours apart^25^. Control mice received 4 doses of sterile saline at 2-h intervals (Table 1). Three weeks after lesioning, fecal pellet output, faecal water content and colonic bead expulsion were assessed in each mouse before euthanasia. This dose (> 40 mg/kg) and time-frame have previously been shown to which has been shown to cause a 50% reduction in nigral neurons^25^.

### Rotenone lesioning

Female WT (n = 21) and A53T (n = 8) mice aged 12 weeks received rotenone (30 mg/kg) daily via oral gavage for 28 days (between 9.00–10.00 AM AEDT) (Table 1). This dose of oral rotenone and duration of treatment has been shown to induce specific nigrostriatal DA neurodegeneration and marked reduction in endurance time on the rota‐rod in C57bl/6 mice^26^. Rotenone was suspended in a solution of 1.25% (v/v) chloroform (Sigma Aldrich, St Louis, USA) and 1% (w/v) carboxymethyl cellulose sodium salt (Sigma Aldrich, St Louis, USA). The total volume of solution delivered was 0.05 mL/10 g of bodyweight. A separate cohort of WT (n = 18) and A53T (n = 6) mice received vehicle solution only (1.25% (v/v) chloroform in 1% (w/v) carboxymethyl cellulose)^27^.

### Fecal pellet output

Fecal pellet output testing was performed as previously described^14^. Briefly, mice were moved from their home cages to individual clean cages containing no bedding and had no access to food and water. The fecal pellets of each animal were collected in pre-weighed tubes every 15 min for 1 h after placement in the new environment. Water content in feces was measured by drying the pellets overnight at 65 °C in an oven and reweighing the tubes. The % water content was calculated using the following equation: [(stool wet weight − stool dry weight)/(stool wet weight)] × 100^14^.

### Bead expulsion test

Immediately following the fecal pellet output testing, mice were subjected to the bead expulsion test as previously described^14^. Mice were lightly anesthetized with isoflurane (2%) to allow insertion of a bead (3 mm in diameter) into the distal colon 2 cm from the anus. Bead insertion was accomplished using a flexible plastic rod to avoid tissue damage. Following bead insertion, mice were placed in individual cages to recover from anesthesia. The time taken from insertion to expulsion was recorded to the nearest second^14^.

### Tissue collection and processing

Mice were anesthetized by intraperitoneal injection with a mixture of ketamine/xylazine (100 mg/kg ketamine and 10 mg/kg xylazine) and transcardially perfused with PBS. Distal colon segments (3–5 cm) were dissected from the abdomen of each mouse and placed in oxygenated PBS (pH 7.2) containing nicardipine (3 µM) (Sigma-Aldrich, Australia) for 10 min to inhibit smooth muscle contraction. They were then cut open along the mesenteric border, cleared of their contents, maximally stretched and dissected mucosa down to expose the myenteric plexus. Tissues were fixed with Zamboni’s fixative (2% formaldehyde, 0.2% picric acid in PBS) overnight at 4 °C. Preparations were cleared of fixative by 3 × 10 min washes with dimethyl sulfoxide (DMSO) (Sigma-Aldrich, Australia) followed with 3 × 10 min washes with PBS. Fixed tissues were stored at 4 °C in PBS for a maximum of 5 days.

### Immunohistochemistry

Wholemount immunohistochemistry was conducted as previously described^14^, colon preparations were incubated with human anti-Hu (1:2000; a gift from Dr Miles Epstein) and sheep anti-nNOS (1:2000; a gift from Dr. Piers Emson) antibodies, overnight at 4 °C. Samples were then washed (3 × 10 min) in PBS before incubation with donkey anti-human Alexa 594 and donkey anti-sheep 488 secondary antibodies, respectively (Molecular Probes, Eugene, OR, USA), for 2 h at room temperature. The tissue was then washed (3 × 10 min in PBS) and incubated with Hoeschst 33258 solution (10 μg/ml Bisbenzimide-Blue in distilled water; Sigma-Aldrich, Sydney, NSW, Australia) for 5 min. Finally, the tissue was again washed before mounting onto glass slides and cover slipped using mounting medium for fluorescent tissues (Dako, Carpinteria, CA, USA).

### Image analysis

The average number of Hu+ cells per area and proportion of nNOS+ cells were quantified in a minimum of six randomly chosen images per preparation (~ 200 to 400 cells/image), from images captured with a 10 × air objective using the AxioImager.Z1 microscope (Carl Zeiss, Sydney, NSW, Australia) and processed using the multipoint tool in image J software (NIH, MD, USA). Ganglia, defined as clustered Hu+ cells less than 1 cell body width apart (~ 20 µm), were traced in image J software using the freehand tool. The number of Hu+ cells per ganglia, and the number of ganglia per area were quantified using the multipoint tool. Cell body size was quantified in 30–50 cells per wholemount preparation from images captured with a 40 × oil objective using Image J software. The freehand tool was used to trace the profile area of each Hu+ nerve cell body, presented in µm^2^.

The proportion of Hu+ translocation was quantified in approximately 100–200 cells per preparation were counted from images captured with a 40 × oil objective. The number of cells displaying visible translocation of Hu into the nucleus were quantified and calculated to the total number of Hu+ cells per image.

The ratio of Hu+ translocation was quantified using Image J software in approximately 100 cells per preparation captured with a 40 × oil objective. The nucleus and cytosol of each cell were separately traced using the freehand tool and mean grey value of each measured. Data is presented as the mean grey value in the nucleus over the mean grey value in the cytosol.

All images were analyzed by an investigator blinded to the genotype/treatment of the mice.

### Statistical analysis

Data were assessed using one-way ANOVA with Tukey’s multiple comparison test or a Welch’s two-tailed t test. Analyses were performed using Graph Pad Prism (Graph Pad Software Inc., CA, USA). Data are presented as mean ± standard error of the mean (SEM). Value differences were considered statistically significant at P < 0.05.

This study was carried out in compliance with the ARRIVE guidelines.

## Results

### A53T transgenic and rotenone-treated mice show a decreased neuronal density within the colon

**Figure 1:**
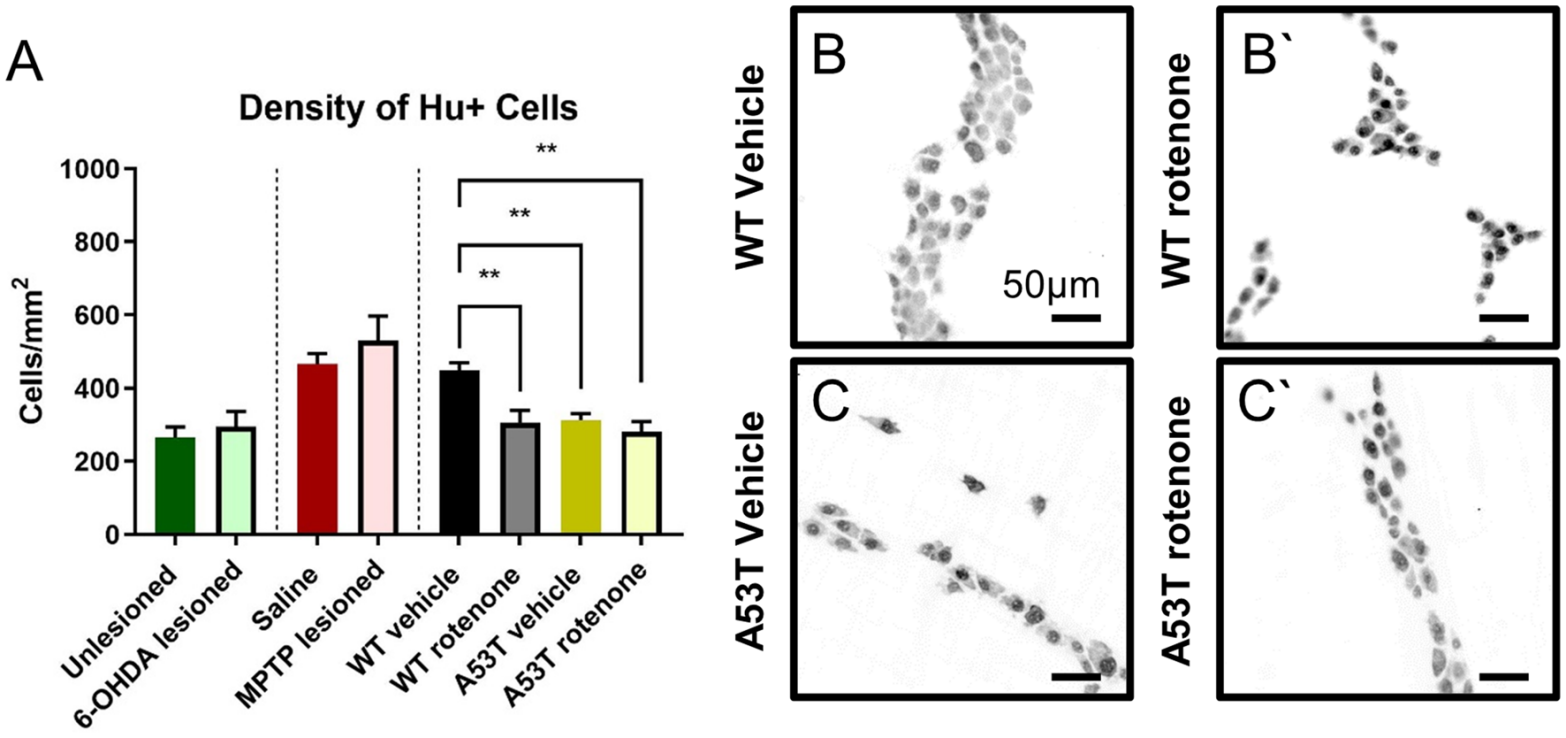
The density of Hu+ neurons per area is reduced in A53T transgenic and rotenone-treated mice, but not in 6-OHDA- and MPTP-treated mice. The number of Hu+ myenteric neurons per mm^2^ was reduced following rotenone treatment in both WT and A53T mice, and A53T vehicle-treated mice had fewer myenteric neurons relative to WT **(A)** Representative photomicrographs of Hu+ neurons in the colon of WT vehicle-treated **(B)**, WT rotenone-treated **(B’)**, A53T vehicle-treated **(C)**, and A53T rotenone-treated **(C’)** mice. Scale bar = 50 µm. Data presented as mean ± SEM. ∗∗P < 0.01 significantly different to relevant vehicle.

To quantify and compare enteric neuronal loss across mouse PD models, the density of occurrence of Hu-immunoreactive (IR) myenteric neurons was assessed. No change in the number of Hu-IR myenteric neurons was observed when comparing 6-OHDA- and sham-lesioned swiss mice (295.3 ± 42.1 neurons/mm^2^ and 265.8 ± 28.4 neurons/mm^2^, respectively, Fig. 1A) or MPTP-lesioned and saline-treated C57Bl/6 mice (531.6 ± 65.6 neurons/mm2 and 465 ± 28.4 neurons/mm^2^, respectively, Fig. 1A). However, significantly fewer Hu-IR neurons occurred in A53T vehicle-treated mice compared to vehicle-treated WT (A53T: 313.2 ± 18.4 neurons/mm^2^, WT: 449.8 ± 19.8 neurons/mm^2^, P < 0.001, Fig. 1B,B’). Treatment with rotenone also significantly reduced the number of Hu-IR neurons in the distal colon of both WT (306.4 ± 33.2 neurons/mm^2^, P < 0.006) and A53T mice (282.4 ± 27.2, neurons/mm^2^ P < 0.001) when compared to vehicle-treated WT mice (449.8 ± 19.8 neurons/mm^2^, Fig. 1C,C’, Table 2).

**Table 2:** Summary of enteric neuropathy in the colon and gut phenotype across mouse models of PD.

	Enteric neuropathy							Gut phenotype		
	Hu+ /area	Hu+ /ganglia	Ganglia/area	Cell body size	%nNOS	%Hu+ translocation	Ratio Hu+ translocation	Bead expulsion	FPO	FWC
6-OHDA vs unlesioned	↔	↓*	↑*	↔	↓**	↔	↔	↔	↔	↔
MPTP vs saline	↔	↔	↔	↔	↔	↑*	↔	↔	↑*	↔
A53T vs WT	↓**	↔	↔	↑*	↔	↔	↔	↔	↔	↔
A53T ROT vs A53T vehicle	↔	↔	↔	↔	↔	↑*	↔	↑*	↓*	↔
WT ROT vs WT vehicle	↓**	↔	↔	↔	↔	↔	↔	↔	↔	↔

*P < 0.05, **P < 0.01.

### 6-OHDA lesioned mice possess fewer neurons per ganglion but more ganglia per area

**Figure 2:**
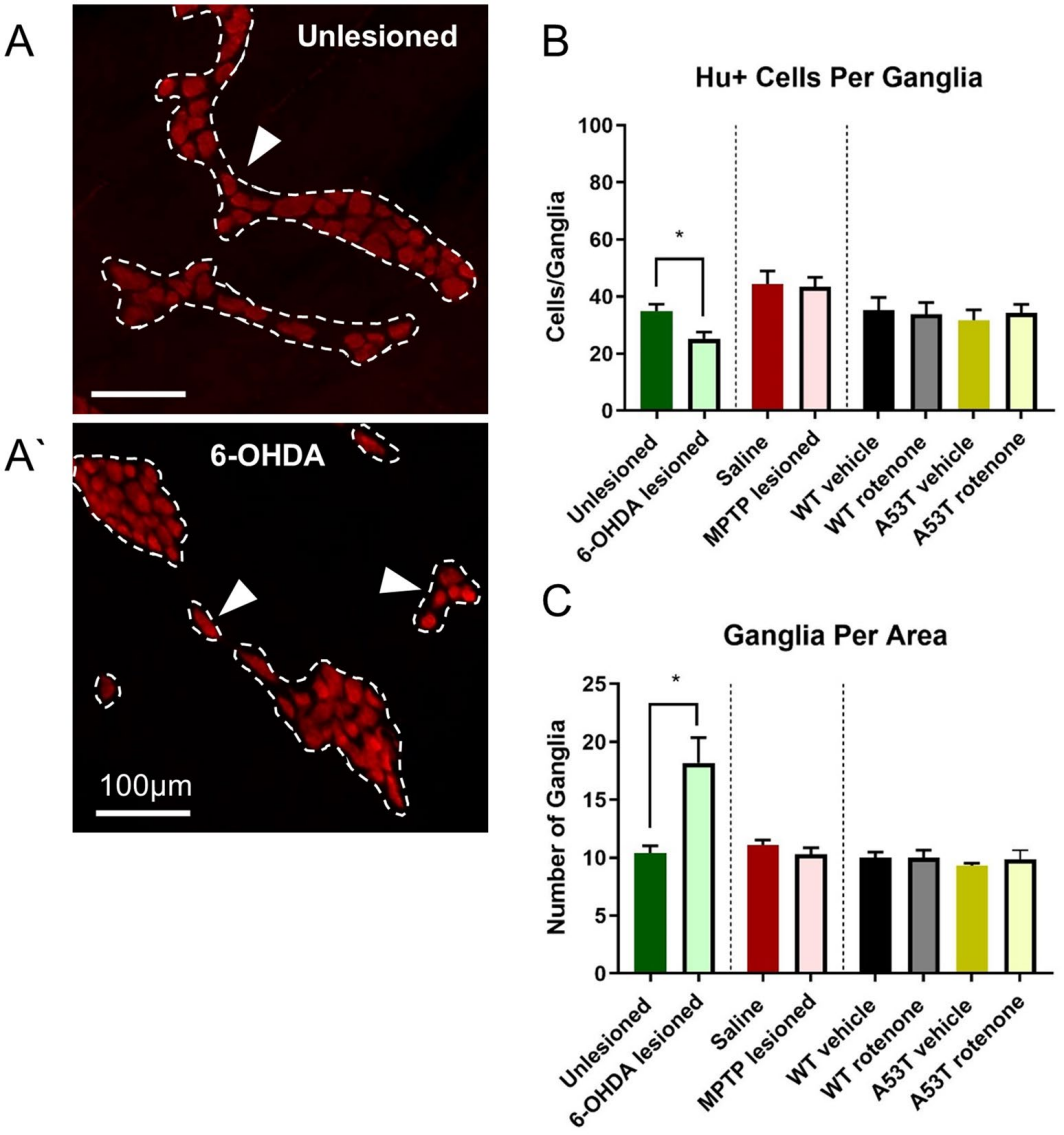
The number of Hu+ cells per ganglia is reduced, but number of ganglia per area is increased following 6-OHDA dosing. Representative photomicrographs of Hu+ myenteric ganglia in unlesioned **(A)** and 6-OHDA lesioned **(A’)** mice, depicting significantly reduced Hu+ cells per ganglia **(B)** and significantly increased ganglia per area in the colon **(C)** of 6-OHDA lesioned mice. Scale bar = 100 µm. Data presented as mean ± SEM. ∗P < 0.05 significantly different to relevant vehicle.

To determine whether the number of neurons per ganglion and ganglia per area was altered in different PD models, the number of Hu-IR neurons per ganglion and number of ganglia per area was assessed. 6-OHDA lesioned mice had significantly fewer Hu-IR neurons per ganglion than unlesioned controls (6-OHDA: 25.2 ± 2.3 neurons/ganglion, Sham: 34.9 ± 2.5 neurons/ganglion, P < 0.02, Fig. 2A,A’, Table 2), however 6-OHDA mice also had a higher density of ganglia per area when compared to unlesioned controls (6-OHDA: 18.2 ± 2.2 ganglia/are, Sham: 10.4 ± 0.6 ganglia/area, P < 0.01, Fig. 2C, Table 2). No change in the number of Hu-IR neurons per ganglion was observed following MPTP treatment (saline: 44.3 ± 4.7 neurons/ganglion; MPTP: 43.6 ± 3.1 neurons/ganglion), rotenone treatment (WT rotenone: 33.8 ± 4.1 neurons/ganglion; A53T rotenone: 34.3 ± 3.0 neurons/ganglion), or in untreated A53T mice and their WT controls (A53T: 31.7 ± 3.6 neurons/ganglion, WT: 35.3 ± 4.4, Fig. 2B, Table 2). Similarly, no change in the number of ganglia per area was observed following MPTP treatment (saline: 11.1 ± 0.4 ganglia/area; MPTP: 10.3 ± 0.6 ganglia/area), rotenone treatment (WT rotenone: 10.0 ± 0.7 ganglia/area; A53T rotenone: 9.8 ± 0.8 ganglia/area), or in untreated A53T mice and their WT controls (A53T: 9.3 ± 0.2 ganglia/area, WT: 10.0 ± 0.5 ganglia/area, Fig. 2C, Table 2).

### The ganglia of A53T transgenic mice possess neurons with larger soma sizes

**Figure 3:**
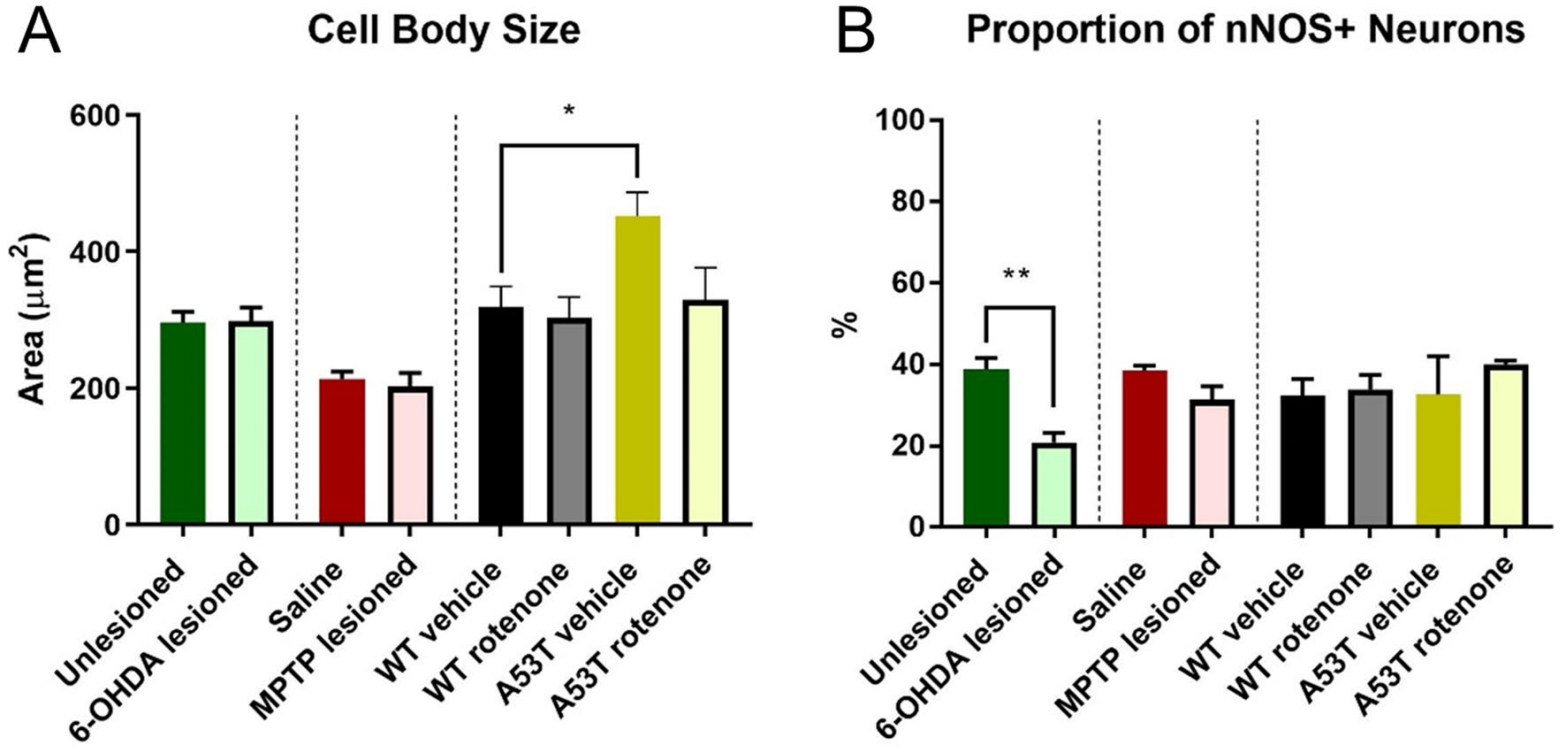
A53T mice have larger soma, and 6-OHDA-lesioned mice have a reduced proportion of nNOS+ neurons. Cell body size in the myenteric plexus of the colon was significantly greater in A53T vehicle-treated mice relative to WT vehicle-treated mice **(A)**. The proportion of nNOS-IR myenteric neurons in the colon was significantly reduced in 6-OHDA-lesioned mice relative to unlesioned mice **(B)**. Data presented as mean ± SEM. ∗P < 0.05 significantly different to relevant vehicle.

To determine whether cell body/soma profile size was altered in the different PD models, individual cell bodies were traced in ImageJ and the area quantified and compared across groups. No difference in soma size was observed when comparing 6-OHDA-lesioned (299.1 ± 26.5 µm^2^) vs unlesioned mice (296.6 ± 15.2 µm^2^) or MPTP-treated (202.5 ± 19.9 µm^2^) vs saline-treated mice (213.7 ± 10.6 µm^2^, Fig. 3A). However, vehicle-treated A53T mice (452.3 ± 34.9 µm^2^, P < 0.03) had significantly larger cell bodies when compared to vehicle-treated WT mice (319.4 ± 29.9 µm^2^, Fig. 3A). Treatment with rotenone had no effect on cell body size in either WT or A53T mice (Table 2).

### 6-OHDA treatment decreased the proportion of nNOS-IR neurons

To determine whether there was any effect on inhibitory motor neurons in the myenteric plexus, the proportion of nNOS-IR neurons to total number of Hu-IR neurons in the myenteric plexus was assessed. 6-OHDA-lesioned mice had a significantly lower proportion of nNOS-IR neurons than unlesioned controls (6OHDA: 20.9 ± 2.3%; Sham: 38.7 ± 2.8%, P < 0.001, Fig. 3B, Table 2). No change in the proportion of nNOS-IR neurons was observed following MPTP treatment (saline: 38.5 ± 1.8%; MPTP: 31.4 ± 3.2%) or rotenone treatment (WT rotenone: 36.3 ± 3.6; A53 rotenone: 40.1 ± 0.9%). Nor was any change found when comparing A53T vehicle (32.4 ± 9.4%) and WT vehicle mice (32.4 ± 4.0%, Fig. 3B, Table 2).

### A53T transgenic mice have a higher degree of Hu+ translocation than WT mice

**Figure 4:**
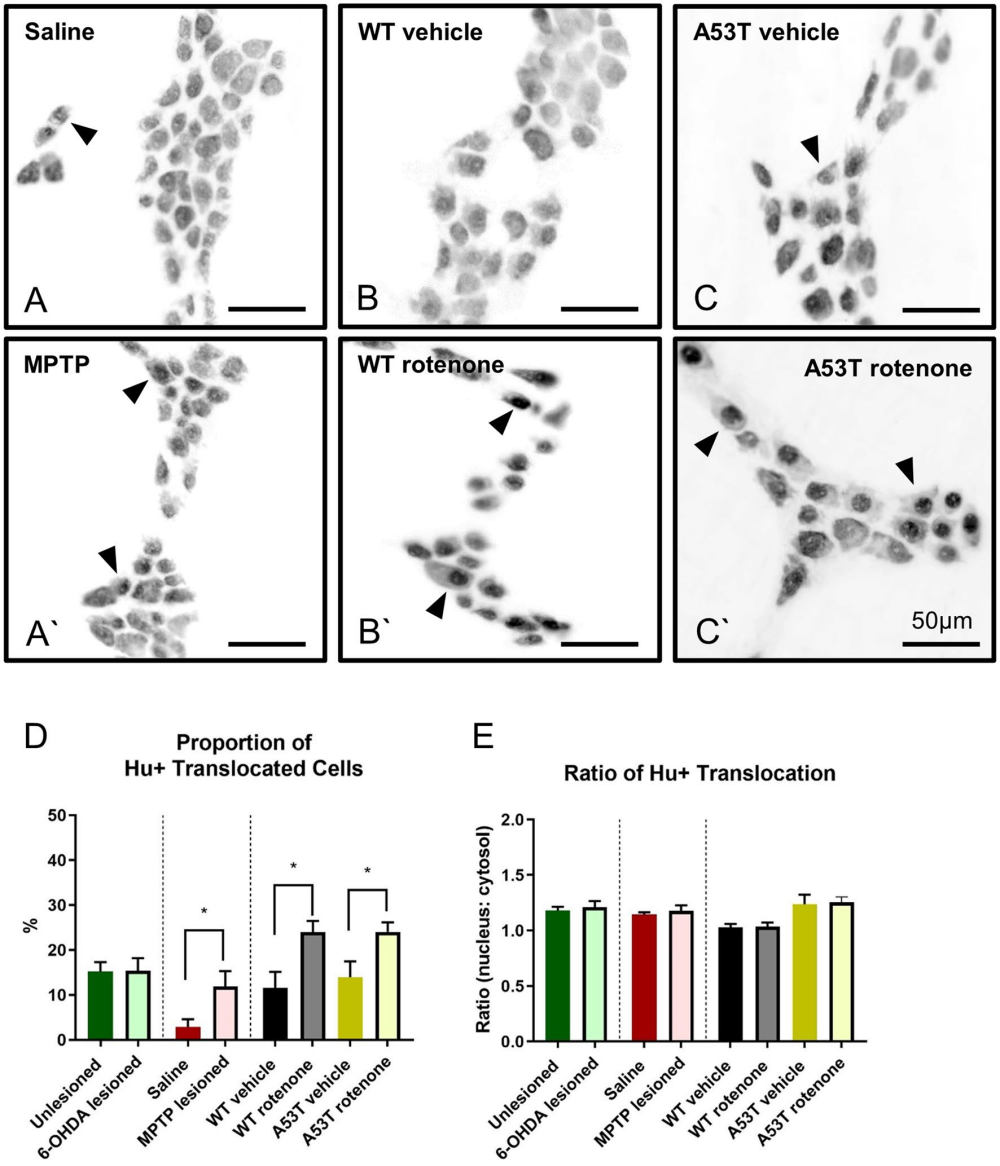
MPTP and rotenone increase the proportion of Hu+ translocated cells. Proportion of Hu+ translocated myenteric neurons in the colon was significantly increased in MPTP-lesioned (A’) WT mice relative to saline WT controls **(A)**, and in both WT **(B’)** and A53T **(C’)** rotenone-dosed mice compared to their vehicle controls (**B**,**C**, respectively). No difference in proportion of Hu+ translocation was found in 6-OHDA-lesioned vs. unlesioned WT mice **(D)**. No difference in the ratio of Hu+ nuclear translocation was found in either 6-OHDA-, MPTP- or rotenone-treated mice relative to vehicle controls, or in A53T mice relative to WT **(E)**. Scale bar = 50 µm. Data presented as mean ± SEM. ∗P < 0.05 significantly different to relevant vehicle.

Translocation of Hu protein to the nuclei of enteric neurons is a marker of neuronal toxicity/distress^14,28^. To assess whether neuronal toxicity varied in different models of PD, both the ratio and severity in individual nerve cell bodies and the proportion of Hu+ translocated cells were quantified. Proportions of Hu+ translocated cells were significantly increased in MPTP-lesioned mice (11.9 ± 3.4%, P < 0.04) when compared to saline-treated mice (2.9 ± 1.7%, Fig. 4A,A’, Table 2). Similarly, treatment with rotenone increased the proportion of Hu+ cell translocation in both WT (24.0 ± 2.5%, P < 0.02 Fig. 4B,B’) and A53T (24.1 ± 2.1, P < 0.04; Fig. 4C,C’) mice. No change in the proportion of Hu+ translocation was found when comparing 6-OHDA-lesioned (15.4 ± 2.8%) vs unlesioned (sham) mice (15.3 ± 2.5, Fig. 4D, Table 2).

When the ratio of Hu+ translocation was assessed, no significant differences in the density of Hu+ translocation were found across any PD model assessed (data not shown, Fig. 4E).

### Fecal pellet output was altered in MPTP-dosed mice and rotenone-treated A53T mice while intrarectal bead expulsion time was altered only in rotenone-dosed A53T mice

**Figure 5:**
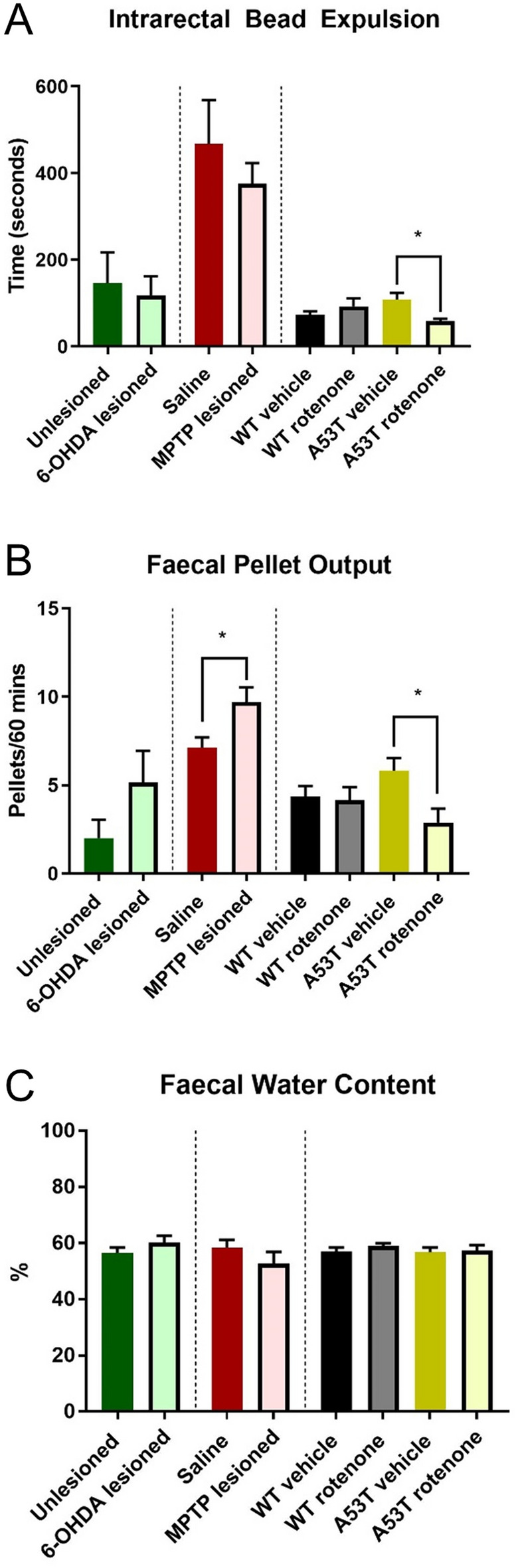
MPTP lesioning and rotenone treatment of A53T mice alters fecal pellet output. Rotenone dosing of A53T mice decreases intrarectal bead expulsion time. No differences were found in intrarectal bead expulsion across any PD model assessed Treatment with rotenone significantly reduced bead expulsion time in A53T mice, but had no effect in WT mice. No difference in bead expulsion times were found in either 6-OHDA- or MPTP-dosed WT mice when compared to their appropriate controls **(A)**. MPTP lesioning increased FPO relative to unlesioned mice, whilst rotenone treatment reduced FPO in A53T mice but not WT mice **(B)**. MPTP lesion also has no impact on FPO relative to saline **(B)**. No differences in FWC were found across all PD model assessed **(C)**. Data presented as mean ± SEM. ∗P < 0.05 significantly different to relevant vehicle.

To determine whether changes at the level of the myenteric plexus were associated with functional consequences, intrarectal bead expulsion time, FPO, and FWC were examined. Treatment with rotenone significantly reduced bead expulsion time in A53T mice (vehicle: 108.1 ± 15.7 s; rotenone: 58.1 ± 5.0 s, P < 0.007, Fig. 5A, Table 2) but had no effect in WT mice (Fig. 5A). No difference in bead expulsion times were found in either 6-OHDA- or MPTP-dosed WT mice when compared to relevant controls (Fig. 5A). FPO was not significantly different following 6-OHDA lesioning (unlesioned: 2.0 ± 1.1 pellet; 6-OHDA lesioned: 5.1 ± 1.8 pellets), but was increased following MPTP lesioning (saline: 7.1 ± 0.6 pellets; MPTP: 9.7 ± 0.9 pellets, P < 0.01, Table 2), and reduced following rotenone treatment in A53T mice (A53T vehicle: 5.8 ± 0.7; A53T rotenone: 2.9 ± 0.8, P < 0.01) but not WT mice (4.4 ± 0.6 pellets; 3.8 ± 0.7 pellets) (Fig. 5B). No difference in FPO was found when comparing vehicle-treated WT and A53T mice (Fig. 5B). No differences in FWC were found across any PD model assessed (Fig. 5C, Table 2).

## Discussion

GI dysfunction is a common non-motor symptom of PD. Whilst in the human condition GI dysfunction is present in 80–90% of patients and has been associated with aggregated α-syn and neuronal loss in the ENS, reports of GI symptoms in animal models of PD are notoriously variable, and the degree to which pathology in the ENS contributes is GI symptoms remains unclear. The current study aimed to compare the level of enteric neuropathy and degree of GI dysfunction across several commonly used mouse models of PD. We demonstrated that all models investigated present some degree of enteric neuropathy- whether it be loss of neurons, augmented nuclear translocation of Hu+ protein, or altered expression of nNOS. However, these changes were not always associated with GI dysfunction.

To evaluate enteric neuropathy, we assessed several parameters associated with neuronal toxicity and stress. Loss of nNOS neurons throughout the gut following 6-OHDA administration to the nigro-striatal pathways has consistently been reported^19,29,30^. In rats, 5 weeks following unilateral 6-OHDA lesion of the medial forebrain bundle, a decrease in the proportion of nNOS-IR neurons in the myenteric plexus has been found in the distal ileum and colon. Altered nNOS expression is one of the most common responses to enteric neuropathy and stress^31^. In the ENS, nNOS neurons, in particular, are highly susceptible to oxidative stress. This is, in part, due to the production and close proximity of the free radical nitric oxide (NO), which can directly induce s-nitrosylation of neuronal proteins and form highly damaging peroxynitrites that can oxidize an array of cellular molecules including proteins and DNA^31^. Unlike findings in the current study which demonstrate a reduction in the number of myenteric neurons per ganglion, the total number of myenteric neurons remained unchanged in 6-OHDA treated rats throughout the GI tract in one study^29^, these findings were mirrored in the current study, with no change in the number of neurons per area following 6-OHDA lesion. This may be due to an increase in the number of ganglia per area. Our data shows an increase in the number of ganglia per area following 6-OHDA lesion, thus whilst the number of neurons per ganglia is reduced, the net number of neurons per area remains the same. In another study^30^, the numbers of neurons was decreased in the ileum, but not the distal colon. Similar to the current study, the proportion of nNOS immunoreactive neurons was decreased in the colon^30^.

How exactly 6-OHDA mediates changes at the level of the ENS remains unclear. Previous research indicates that changes observed following central administration of 6-OHDA are likely trans-synaptic effects in pathways from the brain that send signals down the spinal cord to the defecation centers and then to the colorectum^30^, these effects diminish responses both to defecation center stimulation and to ENS stimulation.

Whilst previously it has been found that MPTP induces a physiological deficit in the inhibitory subpopulation of neurons within the myenteric plexus, including nNOS neurons^17^, the present study indicates no significant change. This is in line with previous studies which have shown MPTP-lesioning significantly reduced the number of dopaminergic tyrosine hydroxylase (TH)-immunoreactive and sensory neurofilament M (NFM)-immunoreactive myenteric neurons in the small intestine, but had no impact on inhibitory nNOS-immunoreactive myenteric neurons^16,17^.

Little research has been undertaken to profile enteric neuron degeneration in A53T transgenic mice. However, our previous work demonstrated a significant decrease in the number of myenteric neurons per ganglion in the distal colon of 15 month old A53T transgenic mice when compared to WT mice^14^. Interestingly in the current study, whilst we were unable to detect a change in the number of neurons per ganglion in A53T mice, we found a significant decrease in the number of neurons per area when compared to WT mice. Our data also indicate that the A53T mutation is not associated with altered nNOS-IR neurons, which is in line with our previous study^14^ as well as that of others investigating the distal colon of Thy1‐αSyn transgenic mice, which demonstrated no difference in vasoactive intestinal peptide- (VIP), choline-acetyl transferase- (ChAT), TH- or nNOS-immunoreactive cells/ganglion when compared to WT^32^.

This is the first study investigating the effects of chronic oral rotenone treatment on enteric neuropathy and GI function in A53T transgenic mice, however exposure to other neurotoxins, including paraquat, maneb and rotenone, has been extensively studied in both rats and mice, yielding conflicting results regarding enteric neuropathy throughout the GI tract^33–36^. Results from the current study indicate that treatment with rotenone induces significant neuronal loss and increases the number of neurons exhibiting Hu+ translocation in the myenteric plexus of WT mice, while having no effect on the proportion of neurons expressing nNOS. Our results are consistent with a previous study investigating oral rotenone treatment in C57bl/6 mice (treated for 28 days), which showed no distinct enteric neurochemical phenotype when compared to sham^33^, though the total number of enteric neurons was not quantified. However, when administered via intraperitoneal injection in rats, rotenone has been found to induce a 25% decrease in the density of myenteric neurons in the small intestine at both 3-days and 6-months post-rotenone treatment^35^.

When rotenone was administered subcutaneously in rats conflicting results have been found. In one study, rotenone was found to have no effect on the total number of neurons, number of NOS or VIP neurons, or the TH containing processes in the myenteric plexus of the pylorus, ileum or proximal colon directly after infusion^36^. However, others have shown that subcutaneous injection of rotenone in rats reduced the number of ChAT-, nNOS- and TH-IR neurons in the colon at 6 weeks post-treatment^37^. Therefore, it is clear that species, duration of treatment and route of administration all play significant roles in the development of rotenone-induced enteric neuropathy.

Interestingly, treatment with rotenone in A53T mice had little effect on number of enteric neurons, but significantly increased the proportion of neurons exhibiting Hu+ nuclear translocation compared to vehicle-control A53T mice. Hu proteins regulate mRNA translation and block the effects of destabilizing proteins when localized within the cytoplasm^38^, thus nuclear localization of Hu may be indicative of impending mRNA degradation and cellular toxicity. Translocation of Hu protein to the nuclei of enteric neurons has been demonstrated in a variety of conditions associated with neuronal toxicity/distress including ischemia and chemotherapeutic treatment^14,28,39,40^. Previous research by our group has shown that presence of the mutant A53T transgene alone is not sufficient to induce nuclear Hu translocation in mice aged up to 15 months^14^. Our data indicates that rotenone treatment is key in induction of nuclear Hu translocation in both WT and A53T mice. It has previously been reported that treatment of A53T mice with rotenone induces compensatory changes in several PD-related proteins, including Parkin and DJ-1^41^. DJ-1, which is a protein sensor that reacts to oxidative stress and protects cells from ROS, was found to be drastically increased in the brains of A53T mice at 2 months post-rotenone treatment^41^. It has been shown that cells with a high level of DJ-1 are resistant to both oxidative stress and to neurotoxins including 6-OHDA whilst low levels of DJ-1 enhance cellular vulnerability to oxidative stress^42^. Whether or not DJ-1 plays a role in mitigating rotenone-induced enteric neuropathy in A53T mice warrants further investigation.

Up to 30% of patients with PD suffer from GI symptoms^5,43^; whilst motility disorders of the stomach (gastroparesis) and colon (constipation) are amongst the most frequent, any portion of the GI tract may be affected^44,45^. In humans, the onset of constipation often precedes the centrally driven motor deficits of PD^8^ and worsen with disease progression. This presentation of PD symptomology is in line with the Braak hypothesis; a widely accepted staging system describing the spread of lewy body pathology from the peripheral to the central nervous system and henceforth in a caudo-rostral fashion^46,47^. In rodent models of PD, conflicting results exist regarding the onset and severity of GI symptoms. Variation in handling and protocols across groups, discrepancies in dosing and duration of treatments and the crude nature of GI functional assays make it difficult to discern subtle changes in gut function, and whether central dysfunction influences GI phenotype.

Previous studies investigating MPTP lesioning in mice have yielded varied results with regards to GI symptoms. Several studies have demonstrated a reduced stool frequency following MPTP treatment^15,16^, whilst others have found that MPTP induced a transient increase in FPO, 2–3 days post-treatment, with no difference detected at 8–10 days post lesioning when compared to sham-treated mice^17^. Whilst Ellett et al. (2016) detected a reduction in fecal pellet output, it is worth noting that this was not calculated relative to baseline (pre-lesioning) but rather to 4 days post-treatment, where is has been shown that FPO is transiently increased^17^. Similarly, Natale and colleagues (2010) calculated fecal pellet output over a 1-week period, starting at 48 h post-lesion; therefore, whether the observed reduction in fecal pellet output is truly sustained following MPTP treatment remains difficult to determine. It is worth noting that whilst both our results and those of Ellet et al. (2016) demonstrated no significant difference in stool weight, thereby indicating that water reabsorption in the colon was likely unaffected by MPTP treatment, Natale and colleagues (2010) reported a 31% reduction in stool weight compared to that of sham-treated mice. Key differences amongst these studies was the dose of MPTP and duration of housing/holding post-treatment; age at start of treatment ranged from 4 to 8 months of age, dose of MPTP spanned from 40 to 60 mg/kg and duration of holding post-treatment varied from 1 to 3 weeks, these factors appear to play a large role in both the degree of enteric neuropathy and the severity of GI symptoms developed.

Whilst our data indicates that 6-OHDA lesioning of mice is not associated with GI dysfunction, previous studies in rats have reported various changes to FPO, intestinal transit time and intrarectal bead expulsion^29,48^. When compared to controls, 6-OHDA-treated rats have shown a significant reduction in daily fecal pellet output at 3 and 4 weeks post-lesioning^29^. Longitudinal muscle contraction and intraluminal pressure are both reduced in the distal colon of 6-OHDA-treated rats^19^. Gastric emptying, intestinal transit, daily FPO and pellet weight have all been found to be significantly reduced at 5 weeks post-lesioning^48^. Additionally, a recent study demonstrated longer intrarectal bead expulsion in 6-OHDA-challengedrats at 4 weeks post-lesioning^30^. Pathologically, both central and peripheral mechanisms contribute to the development of constipation and other GI symptoms^49^, thus whether the effects seen in FPO and bead expulsion are centrally or locally mediated is unknown.

It has previously been shown that transgenic PD mice exhibit GI dysfunction, however the onset and severity of this dysfunction differs across models. Thy1-αSyn mice displayed reduced FPO relative to WT mice as early as 2.5–3 months of age^32^. Similarly, A53T transgenic mice had delayed whole gut transit time, reduced stool frequency and larger stools compared to WT mice at 3 months of age^11^. Contrary to this, a separate study also investigating A53T transgenic mice, found that they do not display reduced stools per hour (faecal pellet output) until 15 months of age, and do not exhibit delayed gastric emptying at 19 months of age when compared to WT mice^13^. These results are comparable with those in our recently published work which demonstrated that A53T mice did not display deficits in intrarectal bead expulsion until 13 months of age, and FPO remained comparable to WT mice until 12 months of age, after which it diminished^14^. In accordance with these data our current study found that A53T transgenic mice display no measurable GI dysfunction when assessed via intrarectal bead expulsion, FPO or fecal water content comparative to WT control mice at ~ 5 months of age.

Treatment with rotenone has also produced varied outcomes in terms of GI symptoms. When injected subcutaneously with rotenone, rats demonstrated a transient decrease in stool frequency between 5 and 10 days post-treatment, however stool frequency was returned to a similar level as sham mice at 25 days post-treatment^36^. Rotenone-injected rats also had higher residual stomach content two hours after a meal, indicating a significant delay in gastric emptying^36^. Similar transient effects have also been noted in mice, with intestinal transit time unchanged at 1-day post-treatment, reduced at 7 days post treatment and unchanged again at 28 days post-treatment in a recent study^50^. In mice, oral delivery of rotenone was found to reduce 1-h stool output in c57bl/6 mice in certain studies^51^ whilst having no impact on gastric emptying, intestinal transit time or intestinal epithelial barrier permeability in other comparable studies^33^. Our current data demonstrated that whilst treatment with rotenone has no effect on GI function in WT mice, it significantly alters both intrarectal bead expulsion and FPO in A53T mice. Whether or not this difference in GI symptomology in WT and A53T mice is reflective of some local compensation in WT mice, or a genotype specific difference in sensitivity to the tests used to assess GI function is unknown. However, our previous work has shown that A53T mice have lower corticosterone levels than WT mice^14^. It has been suggested that alterations in stress hormones, including corticosterone, may affect the normal activity of the dopamine system, and, indeed, corticosterone release is known to play a role in gastrointestinal protection. Increased corticosterone release inhibits gastric erosion in rats, whilst deficiency in glucocorticoid production potentiates functional disorders induced by ulcerogenic stimuli in the gut^52,53^. Thus, differences in corticosterone levels between WT and A53T mice may play a role in differing susceptibility to rotenone-induced damage; this matter requires further investigation.

It has long been known that damage or dysfunction at the level of the ENS can negatively impact GI function. A correlation between enteric neuropathy and GI dysfunction has been demonstrated in several pathologies including Hirschsprung’s disease, achalasia, intestinal neuronal dysplasia, Chagas disease, and severe idiopathic slow-transit constipation^54–57^. Yet, minimal investigation has taken place to understand the relationship between structural changes in the ENS and functional GI consequences in PD. In the current study, changes at the level of the myenteric plexus were seen in all PD models assessed; however, this was not associated with altered GI function in every model. Whilst both 6-OHDA mice and A53T mice exhibited enteric neuropathy when compared to their respective controls, neither displayed any form of measurable GI dysfunction. In contrast, treatment with MPTP and rotenone induced significant Hu+ nuclear translocation which was associated with a dysfunctional gut phenotype. Whilst confounding, these results are somewhat replicative of the human condition, as it has been shown that PD patients suffer varying degrees of enteric neuropathy throughout the gut^58^ and experience different levels of GI dysfunction^20^. The same variability is also seen when comparing central neuron degeneration across rodent models, with different experimental regimens producing varying degrees of nigrostriatal loss and motor dysfunction^59^. This variation in central symptomology has resulted in the suggestion that distinct models may need to be used to replicate early vs. late stage PD, and begs the question, could different models also be utilized to selectively replicate different PD disease stages in the gut?

Acute 6-OHDA and MPTP dosing produces lesions that result in mild to moderate nigral cell loss, with little to no GI symptoms, mimicking early stages of human PD. Whilst chronic dosing with peripheral MPTP and rotenone, consisting of long-term low-dose injections, can produce a more robust lesion^60,61^ alongside GI dysfunction, which may be more pertinent to studying the later stages of PD.

A key advantage of genetic PD models is the slowly progressive accumulation of α-syn which is difficult to replicate chemically. In the current study, A53T transgenic mice treated with rotenone displayed the most pronounced enteric neuropathy and altered colorectal function, encompassing many of the major hallmarks of the human condition and therefore may be the optimal model for further studies investigating GI dysfunction. Though previous research has indicated that α-synuclein pathology is evident in the ENS of A53T mice prior to the onset of central deficits, this was unable to be confirmed in the current study due to the late age at which mice were culled (32 weeks of age).

## Conclusions

In conclusion, animal models with high predictive power are a necessity for translational research. A key strength of this study is the consistency of behavioral testing protocols, tissue processing and analysis across all models investigated. Our results demonstrate that all assessed mouse models of PD displayed enteric neuropathy, with the onset and severity of both enteric neuropathy and GI symptoms heavily influenced by species, age, gender, dose of drug, duration of treatment and time of assessment post-treatment. Thus, both the context of the research, the pathological properties and physical manifestation of the model should be considered before choosing any given model. Whilst our data indicate that A53T transgenic mice treated with rotenone display the most robust GI phenotype, the course nature of the functional assays employed in this study and the impact of stress, from handling, isolation and anesthetic should be considered.

Understanding the role of the ENS in the development of PD-associated GI dysfunction is imperative to the successful identification and prosecution of novel drug targets, and until we have a consistent and reproducible model of the human malady it will remain all but impossible to discover and develop PD therapeutics for GI dysfunction with any level of confidence.

## Abbreviations

α-syn: Alpha synuclein
CNS: Central nervous system
DMSO: Dimethyl sulfoxide
ENS: Enteric nervous system
FPO: Fecal pellet output
FWC: Fecal water content
GI: Gastrointestinal
IR: Immunoreactive
MPTP: 1-Methyl-4-phenyl-1,2,3,6-tetrahydropyridine
nNOS: Neuronal nitric oxide synthase
PD: Parkinson’s disease
SNpc: Substantia nigra pars compacta
TH: Tyrosine hydroxylase
6-OHDA: 6-Hydroxydopamine
